# Discovery of novel PRMT1 inhibitors: a combined approach using AI classification model and traditional virtual screening

**DOI:** 10.3389/fchem.2025.1548812

**Published:** 2025-01-20

**Authors:** Jungan Zhang, Yixin Ren, Yun Teng, Han Wu, Jingsu Xue, Lulu Chen, Xiaoyue Song, Yan Li, Ying Zhou, Zongran Pang, Hao Wang

**Affiliations:** ^1^ School of Pharmacy, Minzu University of China, Beijing, China; ^2^ Institute of National Security, Minzu University of China, Beijing, China; ^3^ Key Laboratory of Ethnomedicine (Minzu University of China), Ministry of Education, Beijing, China

**Keywords:** PRMT1, machine learning, molecular docking, molecular dynamics simulation, molecular hybridization

## Abstract

Protein arginine methyltransferases (PRMTs) play crucial roles in gene regulation, signal transduction, mRNA splicing, DNA repair, cell differentiation, and embryonic development. Due to its significant impact, PRMTs is a target for the prevention and treatment of various diseases. Among the PRMT family, PRMT1 is the most abundant and ubiquitously expressed in the human body. Although extensive research has been conducted on PRMT1, the reported inhibitors have not successfully passed clinical trials. In this study, deep learning was employed to analyze the characteristics of existing PRMTs inhibitors and to construct a classification model for PRMT1 inhibitors. Through a classification model and molecular docking, a series of potential PRMT1 inhibitors were identified. The representative compound (compound 156) demonstrates stable binding to the PRMT1 protein by molecular hybridization, molecular dynamics simulations, and binding free energy analyses. The study discovered novel scaffolds for potential PRMT1 inhibitors.

## 1 Introduction

Protein arginine methylation, which is catalyzed by protein arginine methyltransferases (PRMTs), is the main mechanism regulating the function of eukaryotic cells. This methylation affects epigenetic gene regulation, signal transduction, mRNA splicing, DNA repair, cell differentiation and embryonic development (Yang and Bedford, 2013; Guccione and Richard, 2019).

**FIGURE 1:**
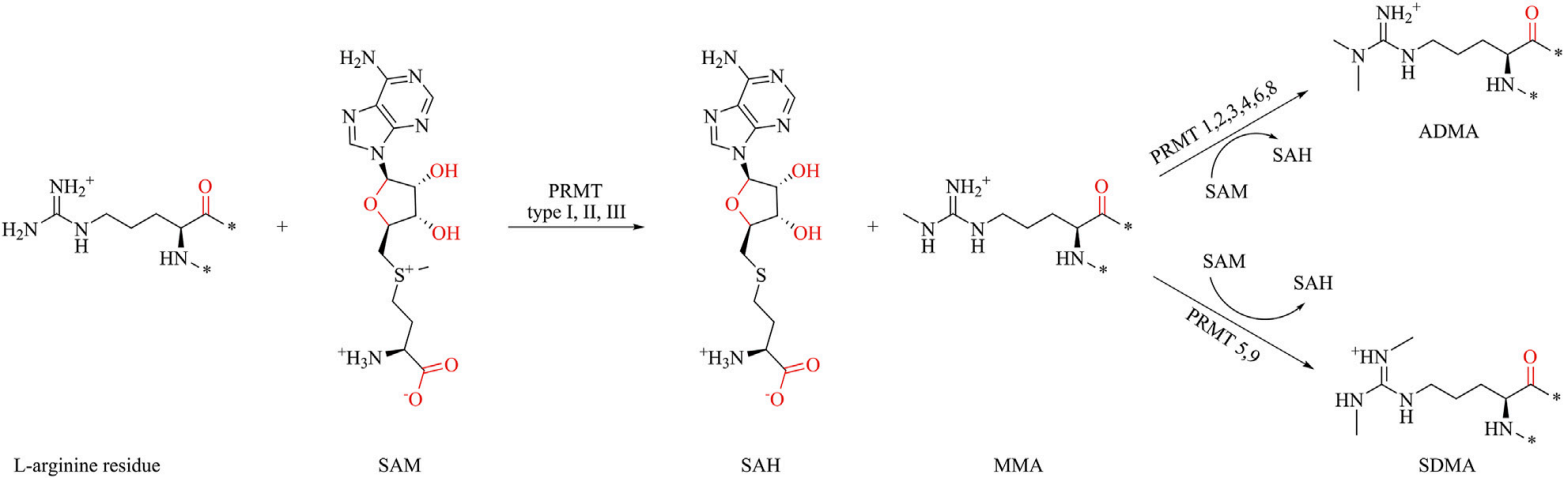
Protein arginine methylation pathways mediated by PRMT types I, II, III and their product specificity.

S-adenosyl-L-methionine (SAM) acts as a methyl group donor, transferring the methyl group to the guanidine nitrogen atom of protein arginine, resulting in the formation of methylated arginine and S-adenosyl-L-homocysteine (SAH) (Figure 1) (Tang et al., 2000b; Passos et al., 2006; Cho et al., 2012; Zhang et al., 2015). PRMTs are classified into three types based on catalytic arginine products. All PRMTs can catalyze the methyl transfer of SAM to arginine, forming monomethylated arginine (MMA). The type I PRMTs catalyze the formation of asymmetric dimethylarginine (ADMA) from MMA, and the type II PRMT catalyzes the formation of symmetric dimethylarginine (SDMA). Type III PRMT only catalyzes the methylation of arginine, and there is no further catalysis. Until now, nine subtypes of PRMTs have been characterized, among which PRMT1, PRMT2, PRMT3, PRMT4, PRMT6, and PRMT8 belong to type I PRMTs; PRMT5 and PRMT9 belong to type II PRMTs; and PRMT7 is the only type III PRMT (Bedford and Clarke, 2009).

The arginine methylation process in *Homo sapiens* (human) cells is primarily mediated by PRMT1, followed by PRMT4 and PRMT5 (Tang et al., 2000a; Bedford and Clarke, 2009). The dysregulation of PRMT1 is associated with the occurrence and development of various diseases, including pulmonary fibrosis (Zakrzewicz et al., 2015), cardiovascular disease (Damiati, 2019), diabetes, nephropathy (Qian et al., 2018), and cancer (Cha and Jho, 2012; Stouth et al., 2017). The overexpression of PRMT1 contributes significantly to the growth, survival, metastasis, and invasion of tumor cells (Baldwin et al., 2012; Yang and Bedford, 2013; Wei et al., 2014). PRMT1 is widely distributed and expressed in human tissues and is highly expressed in cancer cells (Scorilas et al., 2000; Akter et al., 2017). Therefore, PRMT1 is considered a potential target for cancer treatment.

Currently, PRMT1 inhibitors primarily exert their effects through competitive binding with SAM or substrate competition. In addition to the competitive inhibition targeting the functional sites of PRMT1 protein itself, Spring et al. have endeavored to rationally design and synthesize PROTACs for PRMT1. However, the PROTAC developed in that study failed to induce the degradation of PRMT1 (Martin et al., 2024). GSK3368715, the most advanced PRMT1 inhibitor in clinical research, demonstrates potent inhibition of PRMT1 with an IC_50_ of 3.1 nM while showing lower activity against other PRMTs. Unfortunately, despite its potent efficacy, the clinical trial for GSK3368715 was halted due to the “overall benefit–risk profile did not support continuation of the study”, likely because 29% of the patients experienced a thromboembolic event (Fedoriw et al., 2022).

The development and clinical trial of GSK3368715 highlight the immense potential of PRMT1 as a therapeutic target (Junwei et al., 2024). By employing a hybrid approach that integrates artificial intelligence (AI) with computer-aided drug design (CADD), virtual screening can effectively identify and eliminate potential false positives, thereby enhancing the likelihood of identifying compounds with favorable benefit-risk profiles.

**FIGURE 2:**
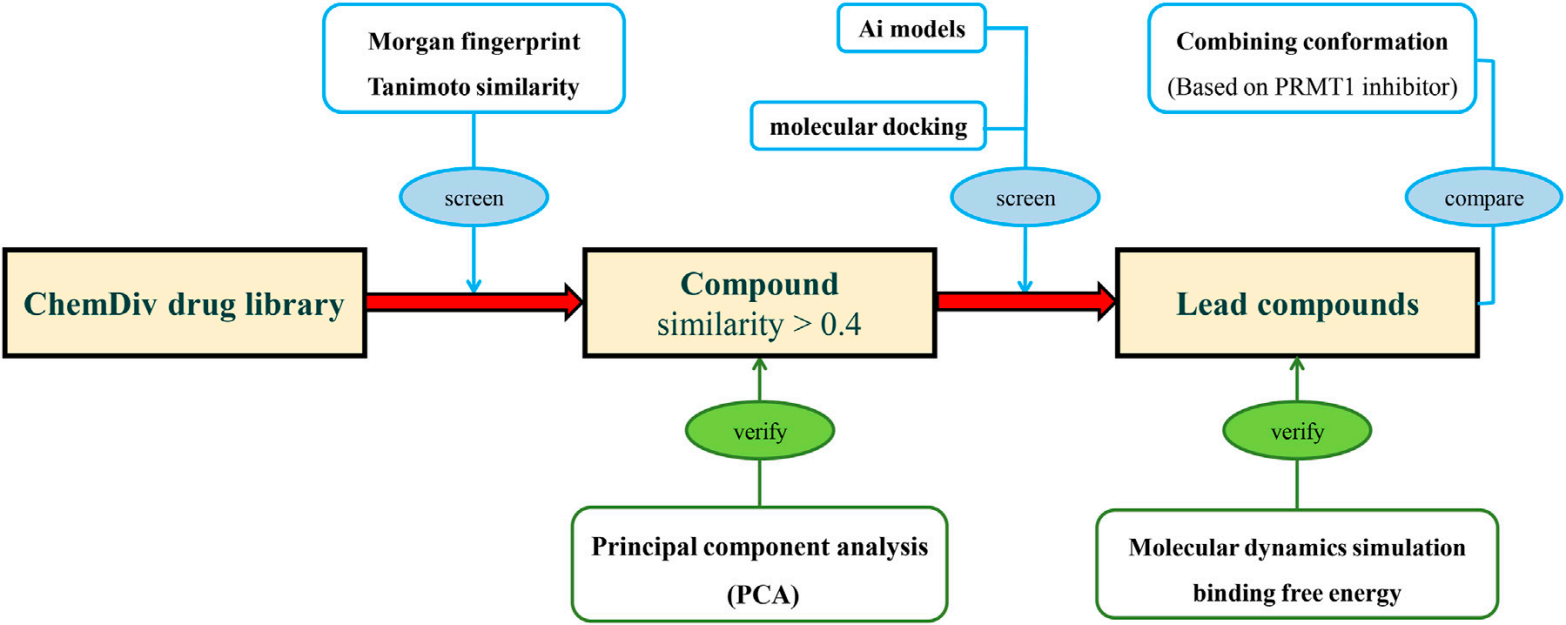
The research process of integrating deep learning, molecular docking, and molecular dynamics simulation for novel PRMT1 inhibitors.

In this study, we trained an AI-based classification model based on PRMTs associated bioactivities from ChEMBL, and designed a screening process to address generalization challenges in AIDD. Initially, compounds were screened for similarity constraints to ensure their resemblance to those used in model construction, and their similarity was validated using principal component analysis (PCA). Subsequently, the classification model and molecular docking were applied to evaluate the constrained molecular database, using known PRMT1 inhibitors as a benchmark for comparison. The compounds most suitable for scaffold modification were further validated through molecular hybridization, molecular dynamics (MD) simulations, and binding free energy calculations (Figure 2).

## 2 Materials and methods

### 2.1 Preprocessing of training and screening datasets

The training dataset was collected from ChEMBL (Davies et al., 2015; Zdrazil et al., 2024). PRMTs associated bioactivities from *H. sapiens* were included in the training dataset to construct the AI screening model. Specifically, the dataset comprised bioactivity data for PRMT1, PRMT3, PRMT4, PRMT5, PRMT6, PRMT7, and PRMT8. The selected data types included IC_50_, Ki, % inhibition, and % activity. Bioactivities without values were excluded, and compounds were annotated based on an activity threshold of 10,000 nM. For ambiguous data, such as those with greater or lesser values, a specific decision-making process (see Supplementary S1.1) was employed with manual assistance.

The SMILE of each compound in the training dataset were proofread from PubChem to obtain standard SMILE data. Salts and other extraneous components were removed to retain only the core compounds as input data. Peptides, macrocyclic molecules, and high molecular weight compounds were removed. To address the imbalance between positive and negative samples in certain datasets, we employed stratified sampling and divided the data into training, validation, and test sets in an 8:1:1 ratio.

The screening datasets were selected from the drug-like libraries collected by our research group, primarily composed of the ChemDiv library, covering 200,000 compounds. Compounds were proofread from PubChem to obtain standard SMILES data. After removing extraneous components, the conformation of each ligand was generated. Autodock Tools 1.5.7 and OpenBabel were used to convert molecular formats (O'Boyle et al., 2011).

The PDB file of PRMT1 was obtained from the Protein Data Bank (PDB ID: 6NT2) (Jumper et al., 2021; Varadi et al., 2021). After removing extraneous components of proteins except SAH, the WHAT IF web server was used to prepare the structure of PRMT1 (Vriend, 1990).

In this study, the PCA relationships of the compounds were calculated using the following parameters: molecular weight (MW), calculated logarithm of the partition coefficient (ALOGP), polar surface area (PSA), hydrogen bond donors (HBD), hydrogen bond acceptors (HBA), rotatable bonds (RB), and aromatic rings (AR). These parameters were calculated using RDKit (Landrum, 2024). The molecular fingerprints of the compounds were constructed using the Morgan fingerprint, which generates a 2048-bit vector representation of a molecule, capturing its structural features based on atom neighborhoods up to two bonds away. The similarity between compounds was calculated using Tanimoto similarity.

### 2.2 Deep learning architecture building

The single-objective and multi-objective classification models in this study were developed using the AttentiveFP from the DeepChem framework (Ramsundar et al., 2019). The model is composed of three parts: the conversion of molecules from SMILES format to graph format using MolGraphConvFeaturizer (Kearnes et al., 2016); the AttentiveFP model proposed by Xiong et al. (2019); and a calculation scheme for AI model performance metrics in the DeepChem framework.

The MolGraphConvFeaturizer is a feature extraction tool designed for molecular graph convolution networks that transforms molecules into graphical representations for use in deep learning models. In this study, node (atom) features were constructed by concatenating various attributes to obtain a total feature length of 30. These attributes include atom type (a one-hot encoded vector for types such as “C”, “N”, “O”, “F”, “P”, “S”, “Cl”, “Br”, “I”, and others), formal charge (integer electronic charge), hybridization (one-hot encoded for “sp”, “sp2”, “sp3”), hydrogen bonding (indicating donor or acceptor status), aromaticity (indicating aromatic ring participation), degree (a one-hot encoded vector for degrees 0–5), and number of hydrogens (a one-hot encoded vector for 0–4 hydrogens attached). Edge (bond) features are similarly constructed, with a total feature length of 11, and include bond type (one-hot encoded for “single”, “double”, “triple”, or “aromatic”), same ring status (indicating if atoms are in the same ring), conjugation (indicating if the bond is conjugated), and stereochemistry (one-hot encoded for stereochemical configuration).

The AttentiveFP model is primarily composed of the K layer for extracting atomic features and the T layer for molecular features, with a fully connected layer for output. In the K layer, GATEConv convolves atomic and edge information, and the T layer pools this information to obtain the overall molecular features. In our model, the T layer is fixed at 2. Hyperparameter optimization was performed using Optuna with 100 trial sets to target the number of K layers, graph feature size, dropout rate, learning rate, and the number of model iterations (Akiba et al., 2019).

The performance of the classification models was evaluated using the metrics AUC-ROC and AUC-PR. The activity data for PRMTs exhibit substantial missing content, as each compound is typically tested against only one or two target proteins. However, in DeepChem calculations, unannotated data are usually treated as negative. This approach conflicts with the target similarity of PRMTs. Therefore, we employed a mask to filter out invalid data, ensuring that only valid data were used in the computations.

### 2.3 Conformation generation by docking

Molecular docking studies were conducted using AutoDock Vina 1.2 (Trott and Olson, 2010; Eberhardt et al., 2021). The methylated arginine site was chosen as the active site and was defined as a 20 × 20 × 20 Å cube box. The docking pocket centers were set as follows: with the SAH centered at x = 8.23, y = 36.627, and z = 43.529; without SAH centered at x = 12.473, y = 30.924, and z = 43.529. The exhaustiveness was set to 64. For other parameters, the default settings were used.

### 2.4 Molecular dynamics simulations and free binding energy calculation

Molecular dynamics (MD) simulations were performed using the AMBER99SB-ILDN force field (Lindorff-Larsen et al., 2010) implemented in the GROMACS 2019.6 program (Berendsen et al., 1995; Van Der Spoel et al., 2005; Abraham et al., 2015), and TIP3P as the water solvation model (Price and Brooks, 2004; Lu et al., 2014). The parameterization of molecules was performed with the general AMBER force field (GAFF) by Sobtop (Lu, 2024; Sousa da Silva and Vranken, 2012). A cube box (with a 0.8 nm buffer distance between the box wall and the nearest solute atoms) was created, and periodic boundary conditions were enabled. A water model was added to the container at a density of 1,000 g/L. The water was replaced by sodium and chlorine ions, aiming to electronically neutralize the system. The system was first minimized through the steepest descent minimization approach (Grubmüller et al., 1991). After that, the restricted molecular dynamics simulation was used to release any restraints. In this restricted molecular dynamics simulation, the temperature of the system was slowly increased to 298.15 K by 500 ps. Lastly, the free dynamic simulations were performed using the Verlet algorithm (Grubmüller et al., 1991). The integration step was set at 0.002 ps. The simulations were performed in an isothermal isobaric regime at 298.15 K and under 1 bar of pressure, with temperature and pressure controlled with the V-rescale and Parrinello-Rahman methods (Berendsen thermostat in annealing) (Parrinello and Rahman, 1980), respectively, and PBC (periodic boundary condition) was enabled. The root mean square deviation (RMSD) was calculated for protein–protein and protein–molecule interactions. MD trajectories were viewed using VMD software (Humphrey et al., 1996).

The binding free energy of the protein-ligand complex was performed by the gmx_MMPBSA package using the molecular mechanics/Poisson-Boltzmann (generalized-Born) surface area method (Massova and Kollman, 2000; Valdés-Tresanco et al., 2021).

### 2.5 ADMET predictions

The absorption, distribution, metabolism, excretion, and toxicity properties were determined using ADMETlab3, a software suite comprising models derived from 88 different datasets containing 375,187 molecules. The R^2^ of each model was greater than 0.95, indicating that the prediction result was statistically significant (Dong et al., 2018).

## 3 Results and discussion

### 3.1 Deep-learning model training and evaluation

**FIGURE 3:**
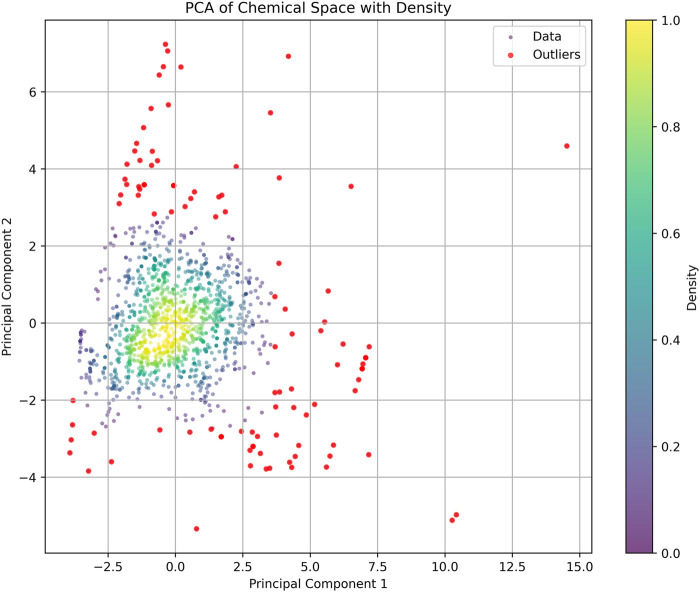
Principal component analysis (PCA) of the training dataset: The scatter plot shows the distribution of compounds based on their properties. Each point represents a compound, with color intensity indicating density. Red point: discrete compounds.

Based on the parameters of molecular weight (MW), calculated logarithm of partition coefficient (ALOGP), polar surface area (PSA), hydrogen bond donors (HBD), hydrogen bond acceptors (HBA), rotatable bonds (RB), and aromatic rings (AR), we constructed a PCA plot to analyze the training dataset (Figure 3). In the PCA analysis, most compounds are concentrated in a central and high-density region, indicated by the yellow and green sections. The distribution of data points forms an approximately circular shape, suggesting that the variance is relatively uniform across the two principal components. Some outliers at the edges of the plot diverge from the main cluster and could unduly influence the model’s training, leading to overfitting or skewed interpretations of the dataset. Outliers can introduce noise and instability in the model, detracting from its ability to generalize across the primary population of compounds. To enhance the robustness of the analysis and improve the quality of the training dataset, we removed these outliers. After this pruning process, the final training set consisted of 1,383 compounds.

We selected the Attentive FP framework as our deep learning model because of its strengths with specific compound types. However, due to the limited number of convolutional layers, this framework is not well suited for handling substructures like polypeptides and macrocyclic compounds. To enhance predictive performance, these structures were excluded from the training dataset. The same exclusion criteria were applied during the final compound screening process.

In the training dataset, activity data for additional PRMT subtypes (PRMT3, PRMT4, PRMT5, PRMT6, PRMT7, and PRMT8) were incorporated to enhance the predictive performance of the single-task model using a multi-task approach. Due to the uneven distribution of positive data and the presence of missing data in the training dataset, the improved area under the precision-recall curve (AUC-PR) metric was selected for model hyperparameter optimization to effectively evaluate the predictive capability of PRMT1. The results of the model hyperparameter optimization are presented in Table 1.

**TABLE 1:** The results of the model hyperparameter optimization.

Parameter	num_layers	Graph _size	Dropout	lr	Epoch
Single-Objective	2	235	0.2578	0.001768	92
Multi-Objective	3	475	0.0705	0.000685	50

**FIGURE 4:**
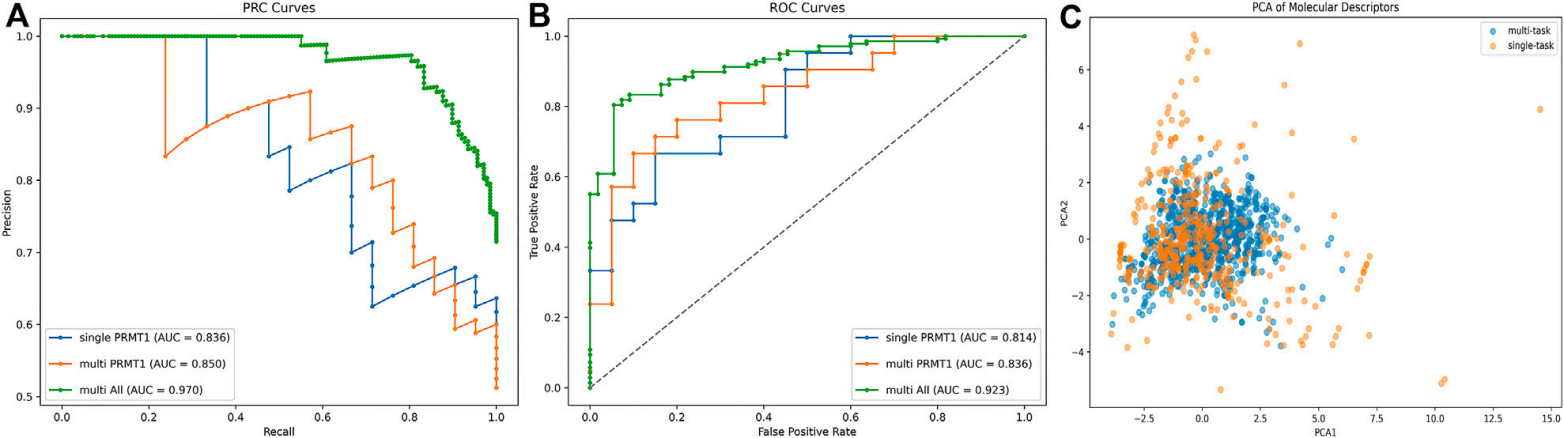
**(A, B)** Comparative analysis of PRC and ROC curves for single-task vs. multi-task classification models in PRMT1 prediction. **(C)** PCA comparison of existing molecules in single objective models and newly added compounds in multi-objective models.

The enhancement of the multi-task model over the single-task model was examined by the area under the receiver operating characteristic curve (AUC-ROC) and AUC-PR metrics (Figure 4). It was observed that the multi-task model improved the predictive ability of PRMT1. For PRMT1 prediction, the AUC-PR increased from 0.836 in the single-task model to 0.850 in the multi-task model, an improvement of 0.014. Similarly, the AUC-ROC increased from 0.814 in the single-task model to 0.836 in the multi-task model, an improvement of 0.022. For all tasks, the multi-task model achieved an AUC-PR of 0.970 and an AUC-ROC of 0.923. Furthermore, the PCA analysis conducted on the newly introduced molecules in the multi-task model, as compared to the existing molecules in the single-task model (Figure 4), revealed that the multi-task model significantly bolstered the robustness of the single-task model. This enhancement mitigated the risk of overfitting to the data of individual tasks and reduced sensitivity to noise and outliers. The results of the AUC-PR demonstrate that the activity data of PRMTs can be generalized and that transfer learning effects can enhance the predictive ability of PRMT1. By accurately recognizing other tasks, the predictive capacity of the PRMT1 model was improved.

### 3.2 Virtual screening of PRMT1 inhibitors

**FIGURE 5:**
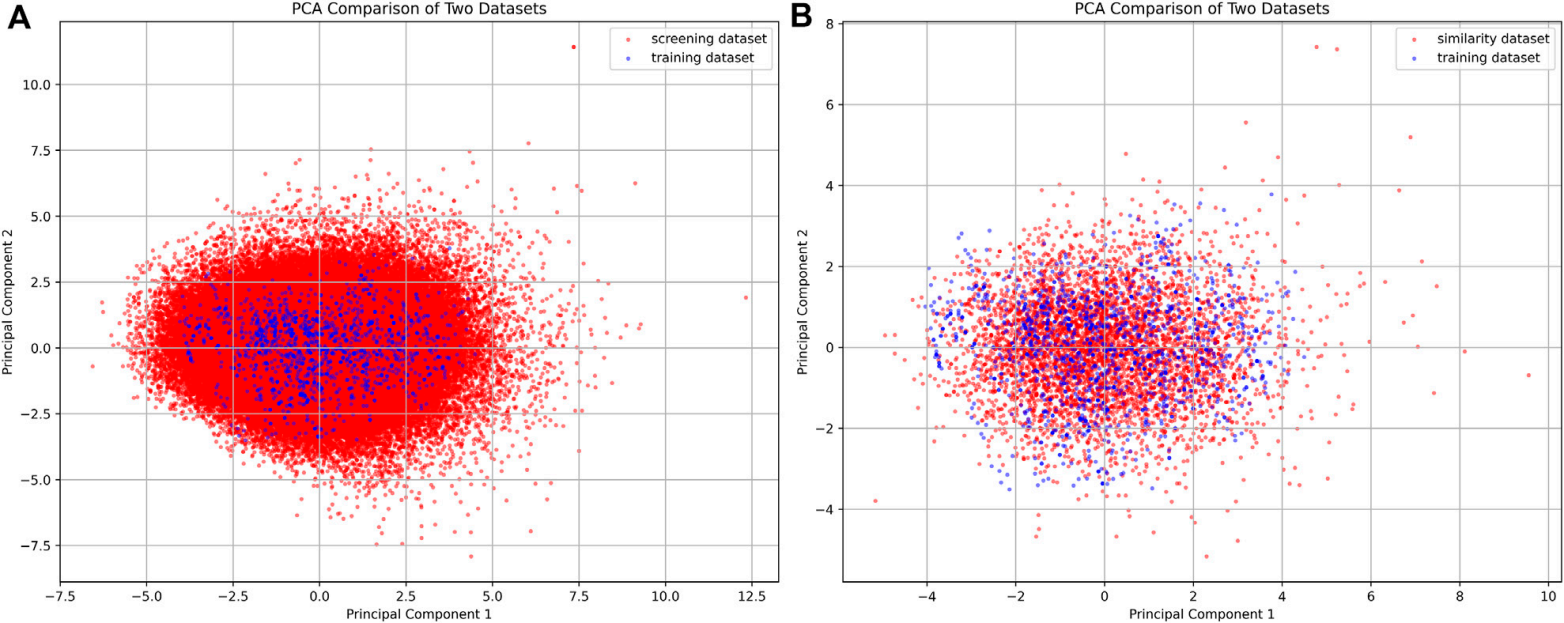
PCA plot of compounds from the filtered compound library and similarity dataset. **(A)** The PCA plot compares the screening dataset (red) with the training dataset (blue). **(B)** The PCA plot compares the similarity dataset (red) with the training dataset (blue).

AI models contain limited generalization abilities because their recognition capabilities depend on the original training dataset. To mitigate the risk of the AI model generating generalization errors, we conducted a screening of compounds using Morgan fingerprint Tanimoto similarity, selecting those with a similarity above 0.4 (Figure 5). PCA was utilized to examine the screening results, revealing that the filtered molecules are well-positioned near the training data. This suggests that the molecules identified through the similarity search have a higher likelihood of being effectively recognized by the AI model, thereby reducing the potential for generalization errors.

**FIGURE 6:**
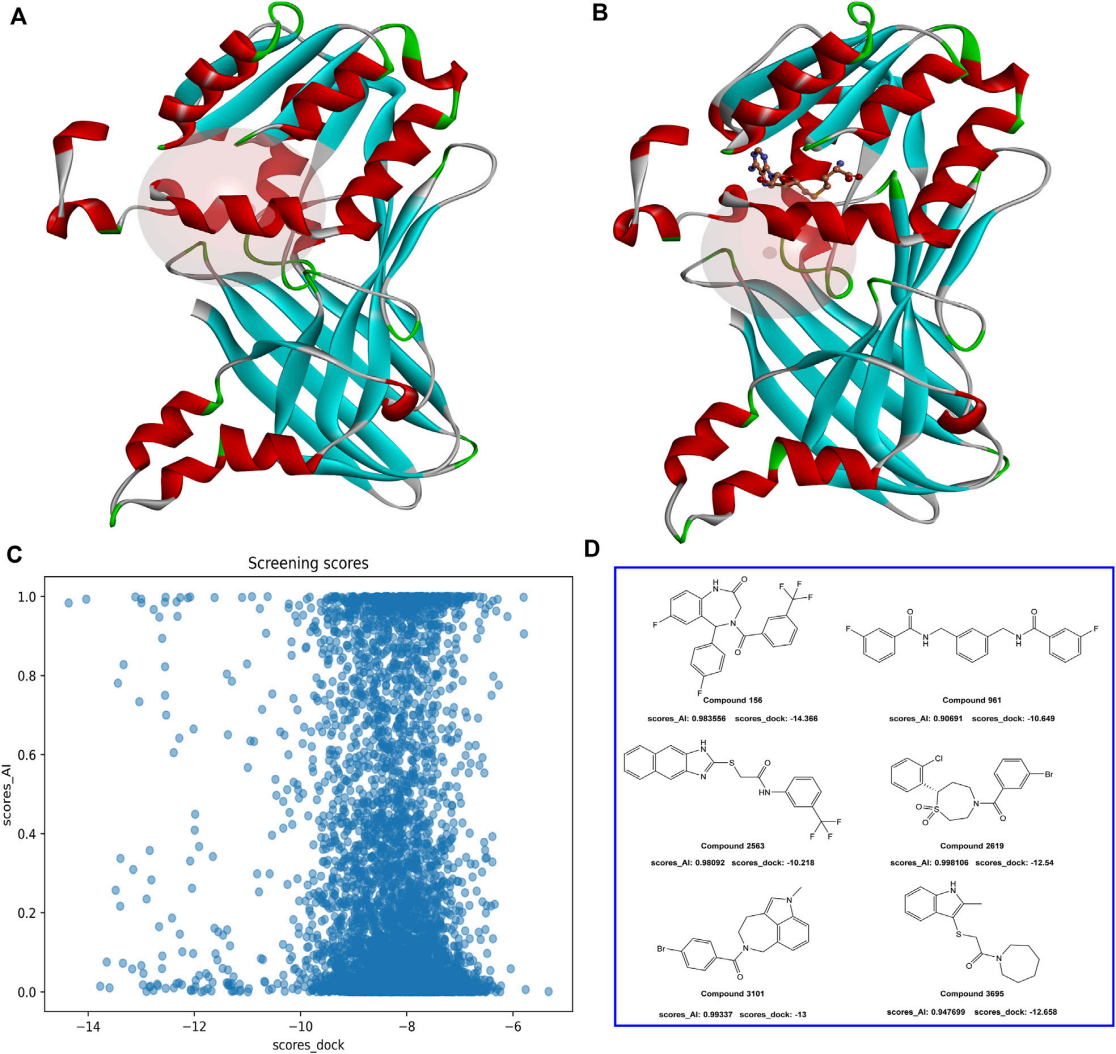
**(A)** PRMT1 inhibitor docking illustration at the natural substrate binding location of SAH. **(B)** PRMT1 inhibitor docking illustration at the SAH-containing arginine binding site. **(C)** The docking and AI model scoring space. **(D)** The top six compounds that satisfy the requirements of AI model scores greater than 0.9 and docking scores less than −10 kcal/mol.

In the virtual screening process, a combined approach of docking and AI screening models was employed to evaluate the compounds from the molecular database generated by the similarity search. During the molecular docking process, we considered the multiple inhibition mechanisms of PRMT1 inhibitors, including substitution at the SAH natural substrate binding site and inhibition at the arginine binding site containing SAH. Accordingly, we performed two docking modes on the PRMT1 protein, each targeting one of the inhibitor binding mechanisms (Figure 6). In parallel, we employed our constructed multi-target classification model for PRMTs during the AI screening process. In this step, we input the compounds in SMILES format into the AI model, targeting the positive PRMT1 scoring as the primary measure for AI-based assessment. We compared the docking scores with the AI model scores. As shown in Figure 6, the evaluated molecules are well-distributed within the scoring space. Molecules with docking scores less than −10 kcal/mol and AI positive model probabilities greater than 0.9 were selected.

**FIGURE 7:**
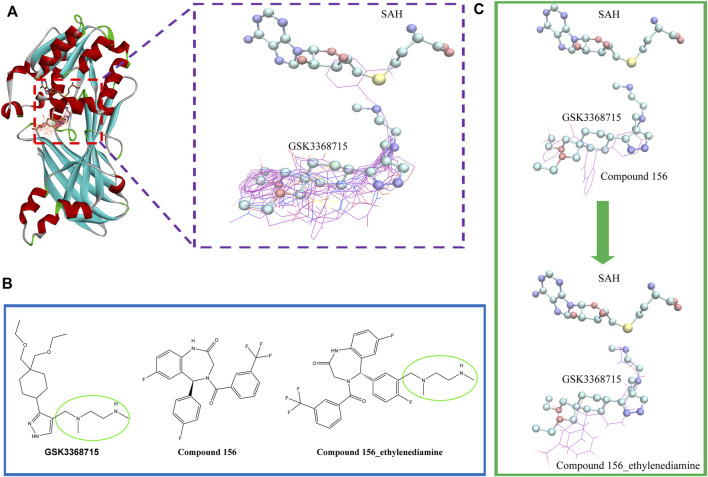
**(A)** Positional relationship between the screened molecule (line), SAH, GSK3368715 (stick), and protein crystal structure (ribbon). **(B)** 2D Structures of compound 156, compound 156-ethylenediamine (compound 156 N), and GSK3368715. **(C)** The positional relationship between compound 156 and compound 156 N with GSK3368715 and SAH.

To investigate the binding conformation of the screened molecule within the PRMT1 protein, we aligned the docking conformation onto the reported type 1 PRMT inhibitors (PDB ID: 6NT2) (Figure 7). It is evident that the screened molecule does not act as an SAH substitute; instead, it primarily binds through the arginine pocket.

Currently, the most effective PRMT1 inhibitors reported in the literature contain a methylene-N-methylethylenediamine moiety attached to a five- or six-membered aromatic or heteroaromatic group (Hendrickson-Rebizant et al., 2024). This structural motif has demonstrated the best activity among those studied so far (Yang et al., 2017; Fedoriw et al., 2019; Wang et al., 2022). Through visual inspection, we identified compound 156 for further investigation. The selection of this compound was primarily due to its docking conformation, where it was found to be the only compound that meets the criteria for molecular hybridization involving the N-methylethylenediamine component.

Molecular hybridization was performed by connecting compound 156 with N-methylethylenediamine, which is the core structural component in PRMT1 inhibitors, to obtain compound 156-ethylenediamine (Figure 7). To assess the binding conformation of compound 156-ethylenediamine, we employed the same docking methodology as previously described for the initial screening. The binding rationalities of compound 156, compound 156-ethylenediamine, and compound GSK3368715 were further validated through MD simulations. We conducted three separate 100 ns simulations, each involving a compound in a ternary complex with SAH and PRMT1.

**FIGURE 8:**
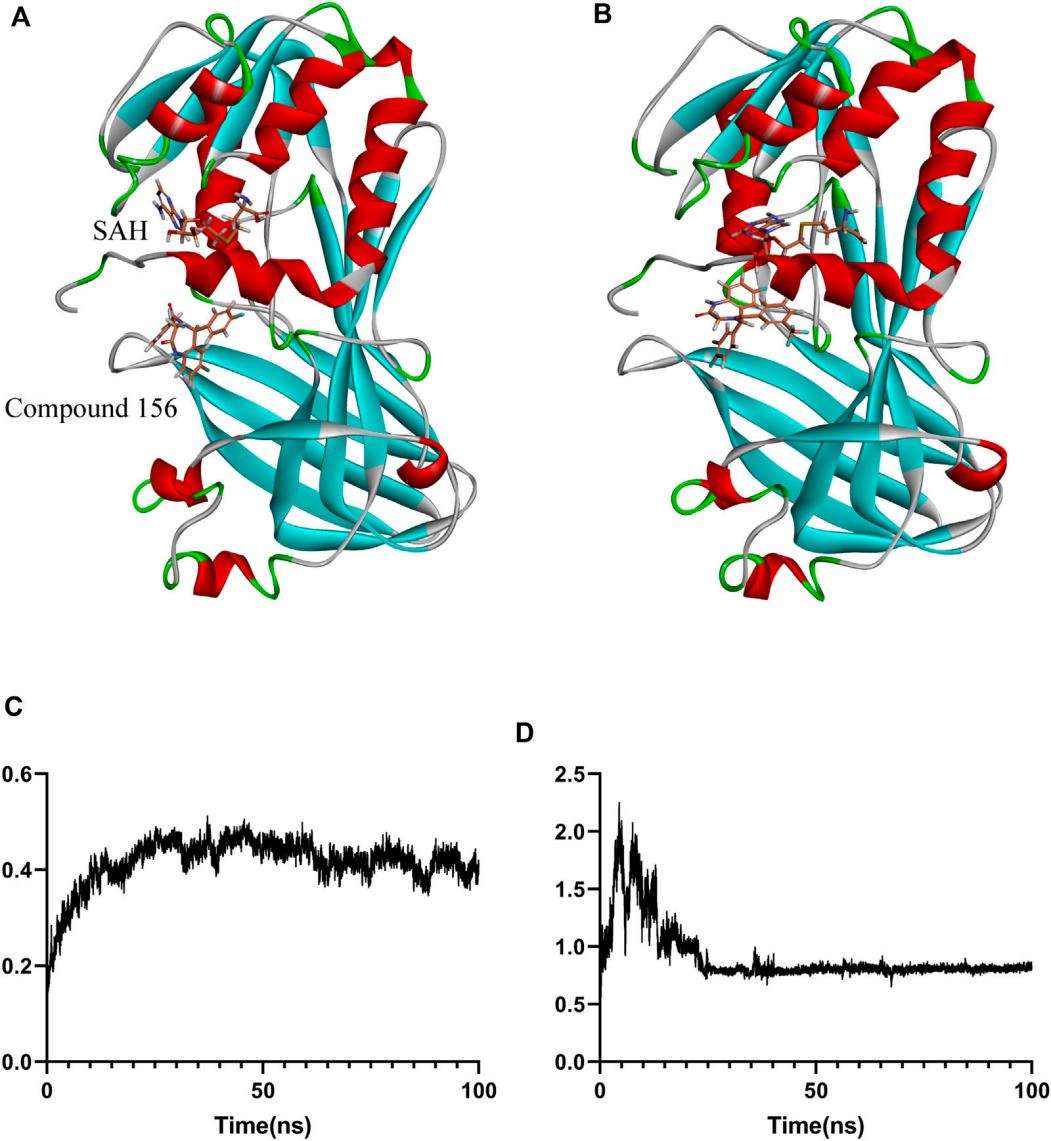
Binding positional changes and RMSD analysis of compound 156: **(A)** Position before simulation; **(B)** Position after simulation; **(C)** RMSD of compound 156; **(D)** RMSD of the protein.

In the simulation of the positive control compound GSK3368715 (derived from the previously reported crystal structure, PDB ID: 6NT2) and the simulation of compound 156-ethylenediamine, the SAH failed to adopt a binding mode analogous to that observed in the crystal conformation. Only the simulation of compound 156 exhibited a relatively stable binding conformation for SAH, which remained near the initial binding site (Figure 8). To ascertain the simulation instability induced by GSK3368715 and compound 156-ethylenediamine, we conducted six replicate experiments for each compound: three iterations were performed on the PRMT1 monomer, while the remaining three were executed on the PRMT1 dimer based on the crystal structure.

For the stable binding compound, as shown in Figure 8, compound 156 and protein underwent conformational changes at the beginning of the simulation. However, after 30 ns, as the protein conformation stabilized, the ligand maintained relative stability throughout the remaining simulation. The binding free energy was calculated using the GBSA method from 80 ns to 100 ns, which showed that the average binding free energy of compound 156 to the SAH and PRMT1 complex was −22.74 ± 0.43 kcal/mol.

For the remaining compounds with unstable binding, the binding free energy was calculated through the GBSA method over the interval of 1 ns–100 ns, revealing that the average binding free energy of compound 156 to the SAH and PRMT1 complex was lower than that of the GSK3368715 control, with values of −22.74 ± 0.43 kcal/mol for compound 156 compared to −36.05 ± 0.47 kcal/mol for GSK3368715. Compound 156-ethylenediamine exhibited comparable efficacy to GSK3368715, with values of −37.07 ± 0.44 kcal/mol, thereby indicating that compound 156-ethylenediamine can effectively bind to the inhibitor site of the PRMT1 protein.

### 3.3 Binding interaction analysis against PRMT1

**FIGURE 9:**
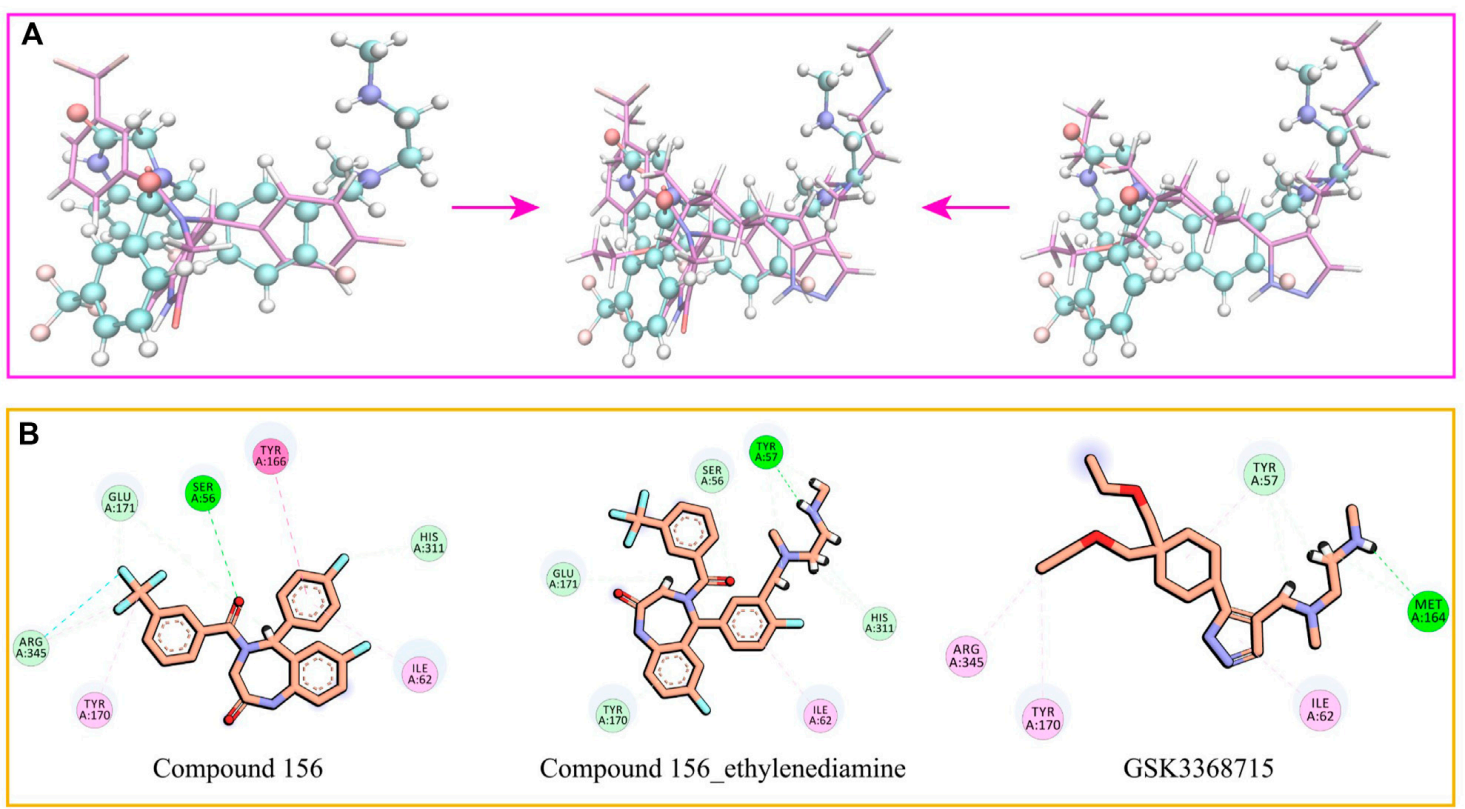
**(A)** Comparative structural analysis of compound 156 and compound 156-Ethylenediamine with GSK3368715. **(B)** Binding interactions of compound 156, compound 156-Ethylenediamine, and GSK3368715 with PRMT1 active site residues.

Drawing upon the results from molecular docking studies, we meticulously investigated and validated the binding interactions of the three compounds with the active site amino acid residues of PRMT1. Through a pairwise positional comparison of the compounds (Figure 9), compound 156-ethylenediamine emerges with a novel conformation, exhibiting slight variances from its two precursor molecules.

In comparison to GSK3368715, compound 156-ethylenediamine preserves the original interacting residues Ile62 and Tyr57. The N-ethylenediamine segment remains situated within its pocket. However, the compound underwent a certain angular displacement, resulting in the forfeiture of hydrogen bonds potentially formed with Met164 while simultaneously facilitating the development of new hydrogen bonds with Tyr57.

Relative to compound 156, compound 156-ethylenediamine has experienced rotation, yet the critical residues interaction Ser56, Ile62, Tyr170, Glu171, and His311 have been retained. Moreover, the benzene ring utilized for molecular hybridization loses its π-conjugated positioning with Tyr166 due to positional dislocation.

The interactions among the residues of the three compounds are detailed in Table 2. Compound 156-ethylenediamine adeptly inherits the interactions of its two source molecules, and as the source molecule, compound 156 reveals a promising potential for binding to the PRMT1 scaffold.

**TABLE 2:** Statistics of residues interacting with PRMT1 protein by three compounds.

Compounds	Hydrogen bonds and Van der Waals forces	Conjugation and hydrophobic interaction
GSK3368715	Tyr57, Met164	Ile62, Tyr170, Arg345
156-ethylenediamine	Ser56, Tyr57, Tyr170, Glu171, His311	Ile62
156	Ser56, Glu171, His311, Arg345	Ile62, Tyr166, Tyr170

### 3.4 ADMET predictions

We employed the ADMETlab 3.0 computational model to forecast the absorption, distribution, metabolism, excretion, and toxicity (ADMET) characteristics of the compound, which are imperative for evaluating its drug-like potential.

In terms of synthetic accessibility, GSK3368715 was assessed as “hard,” whereas compound 156 and compound 156-ethylenediamine were deemed “easy.” The molecules presented herein have been rated as relatively easy to synthesize according to AI scoring.

The compound 156 and compound 156-ethylenediamine improve the subpar Caco-2 permeability and metabolic clearance rates associated with GSK3368715 by retaining the structural framework of compound 156. However, it also inherits the latter’s elevated plasma protein binding affinity.

In predictions of acute oral toxicity in rats, compound 156 and compound 156-ethylenediamine exhibited heightened toxicity. Additionally, all three compounds tested positive for human liver toxicity and genotoxicity, although such toxicities can frequently be alleviated through optimization of the chemical structure.

**FIGURE 10:**
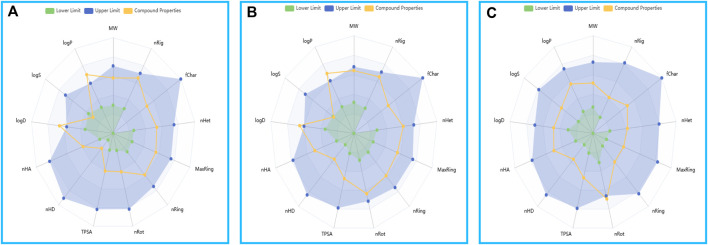
The drug physicochemical property radar map, generated by ADMETlab 3.0. **(A)** Compound 156. **(B)** Compound 156-ethylenediamine. **(C)** GSK3368715.

The ADMET predictions affirmed that the physicochemical properties of the two compounds and GSK3368715 align with the drug-like realm delineated by the ADMETlab 3.0 platform (Figure 10).

## 4 Conclusion

PRMT1, a key protein responsible for arginine methylation, has received extensive research attention. Currently, PRMT-targeted drugs have not successfully completed clinical trials. This suggests that PRMT1 has a sufficient data foundation for AI design and that subtype data can enhance AI model screening capabilities. In contemporary drug development, the combination of virtual screening and AI has become an effective research strategy.

In this study, we employed a hybrid approach that combines AI screening with traditional drug screening processes. To address the challenge of AI models recognizing out-of-distribution data that may lead to hallucination errors, we introduced fingerprint similarity to constrain the similarity between the screened and training compounds. This approach reduces the misjudgment of unrecognized molecules entering the AI scoring phase. Our final PRMT1 screening model achieved an AUC-PR of 0.850, demonstrating its ability to identify positive compounds.

Through AI model screening and molecular docking, we identified compound 156 that can cross-link with the key backbone molecule of the best PRMT1 inhibitor reported in the literature. Molecular hybridization was performed on the six-membered ring at the corresponding position of compound 156, and compound 156-ethylenediamine containing N-methylethylenediamine was obtained. Molecular docking proved that compound 156-ethylenediamine inherited the key structures of both compounds and could interact with the corresponding amino acid residues. And exhibited good stability during molecular dynamics simulations, proving the ability of compounds 156 to bind to the outer pocket of PRMT1 N-methylethylenediamine. The ADMET prediction indicated that the identified compounds possess favorable drug-like properties.

As a lead compound, compound 156 was shown to have the ability to bind to the PRMT1 pocket, and this ability could be transferred to the original optimal skeleton, indicating that it has the potential for further structural modification and selectivity enhancement.

## Data Availability

The original contributions presented in the study are included in the article/Supplementary Material, further inquiries can be directed to the corresponding authors.
