# Supplementary material for: Discovery of novel PRMT1 inhibitors: a combined approach using AI classification model and traditional virtual screening
Source: Front Chem. 2025 Jan 20;13:1548812. doi: 10.3389/fchem.2025.1548812 (PMC11788407; doi:10.3389/fchem.2025.1548812)
Supplement: Supplementary file 1 [file Supplementaryfile1.docx]

Supplementary Material

# Supplementary Data

## Data Classification Standards

**IC50 and Ki Classification**:

If the "Standard Relation" is '=' or '<', and the "Standard Value" is less than 10,000, data is marked as positive (1).

If the "Standard Relation" is '=' or '<', and the "Standard Value" is greater than or equal to 10,000, data is marked as negative (0).

If the "Standard Relation" is '>', and the "Standard Value" is less than 10,000, data is marked for manual review (4).

If the "Standard Relation" is '>', and the "Standard Value" is greater than or equal to 10,000, data is marked as negative (0).

**Inhibition Classification**:

If the "Standard Relation" is '=' or '>', and the "Standard Value" is greater than or equal to 10, data is marked as positive (1).

If the "Standard Relation" is '=' or '>', and the "Standard Value" is less than 10, data is marked as negative (0).

If the "Standard Relation" is '<', and the "Standard Value" is greater than or equal to 10, data is marked for manual review (4).

If the "Standard Relation" is '<', and the "Standard Value" is less than 10, data is marked as negative (0).

**Activity Classification**:

If the "Standard Relation" is '=' or '<', and the "Standard Value" is less than or equal to 90, data is marked as positive (1).

If the "Standard Relation" is '=' or '<', and the "Standard Value" is greater than 90, data is marked as negative (0).

If the "Standard Relation" is '>', and the "Standard Value" is less than or equal to 90, data is marked for manual review (4).

If the "Standard Relation" is '>', and the "Standard Value" is greater than 90, data is marked as negative (0).

**Other Types Classification**:

Data not falling into the above categories is marked for manual review (5).
